# Systematic genomic identification of colorectal cancer genes delineating advanced from early clinical stage and metastasis

**DOI:** 10.1186/1755-8794-6-54

**Published:** 2013-12-05

**Authors:** HoJoon Lee, Patrick Flaherty, Hanlee P Ji

**Affiliations:** 1 Division of Oncology, Stanford University School of Medicine, Stanford, CA 94305, USA; 2 Department of Biomedical Engineering, Worcester Polytechnic Institute, Worcester, MA 01605, USA; 3 Stanford Genome Technology Center, Stanford University, Palo Alto, CA 94304, USA

**Keywords:** Colorectal cancer, Genomics, Genetics, Clinical stage, Metastasis

## Abstract

**Background:**

Colorectal cancer is the third leading cause of cancer deaths in the United States. The initial assessment of colorectal cancer involves clinical staging that takes into account the extent of primary tumor invasion, determining the number of lymph nodes with metastatic cancer and the identification of metastatic sites in other organs. Advanced clinical stage indicates metastatic cancer, either in regional lymph nodes or in distant organs. While the genomic and genetic basis of colorectal cancer has been elucidated to some degree, less is known about the identity of specific cancer genes that are associated with advanced clinical stage and metastasis.

**Methods:**

We compiled multiple genomic data types (mutations, copy number alterations, gene expression and methylation status) as well as clinical meta-data from The Cancer Genome Atlas (TCGA). We used an elastic-net regularized regression method on the combined genomic data to identify genetic aberrations and their associated cancer genes that are indicators of clinical stage. We ranked candidate genes by their regression coefficient and level of support from multiple assay modalities.

**Results:**

A fit of the elastic-net regularized regression to 197 samples and integrated analysis of four genomic platforms identified the set of top gene predictors of advanced clinical stage, including: *WRN*, *SYK*, *DDX5* and *ADRA2C*. These genetic features were identified robustly in bootstrap resampling analysis.

**Conclusions:**

We conducted an analysis integrating multiple genomic features including mutations, copy number alterations, gene expression and methylation. This integrated approach in which one considers all of these genomic features performs better than any individual genomic assay. We identified multiple genes that robustly delineate advanced clinical stage, suggesting their possible role in colorectal cancer metastatic progression.

## Background

Colorectal cancer (CRC) is projected to be the 3rd leading cause of cancer deaths in the United States in 2013 with the mortality primarily a result of metastatic disease [1]. Identifying the genetic and genomic basis of CRC has significant clinical implications. Our understanding of CRC requires identification of the critical “driver” genes that are fundamentally important for CRC development unlike “passenger” genetic aberrations that have no functional relevance to cancer biology [2]. Previous genetic and genomic studies of CRC have identified many of the critical drivers that are important to CRC development [3-7]**.** For example, the cancer genes *APC*, *KRAS* and *TP53* have a high frequency of genetic aberrations in CRC and are known to play an essential role in CRC development [8]. A number of other cancer genes have been identified in CRC and cluster in several biological pathways including those responsible for Wnt signaling [9], RAS/RAF pathway [10] and transforming growth factor β (TGF-β) signaling [11]. While the cancer genes directly responsible for CRC development have been characterized, less is known about which cancer genes delineate advanced versus early stage CRC.

Currently, the metastatic status of CRC is assessed via clinical staging which dictates the choice of therapy and remains the best prognostic indicator for individual CRC patients [12]. Clinical stage is determined by the TNM criteria, where T is assigned by extent of tumor invasion, N represents the number of lymph nodes with metastatic cancer and M represents the presence of metastatic cancer in other organs outside of the colon and lymph nodes. Advanced clinical stage either reflects metastatic cancer spread to the regional lymph nodes around the colon as in stage III or spread to organs outside of the colon or rectum as in stage IV. Advanced (stages III or IV) CRC has a significantly worse prognosis compared to early stage (stages I or II) that is generally considered curable. With the advent of genomic cancer medicine, there is increasing interest in identifying the specific CRC genetic aberrations and related cancer genes that define advanced clinical stage. Identification of these genetic aberrations and their corresponding cancer genes may illuminate the underlying genetics of advanced clinical stage CRC as well as have relevance in the prognostic assessment.

A recent large-scale study by the Cancer Genome Atlas (TCGA) is the most comprehensive CRC genomic survey to date [13]. The TCGA CRC project relied on a combination of next generation sequencing and microarray genomic platforms to characterize different CRC genetic aberration features and the individual affected genes. This project also provides clinical information about the metastatic status of individual patients via clinical stage information. The breadth of the TCGA genomic data sets provides a unique opportunity to consider different categories of genetic aberrations at individual gene resolution that other genomic studies have not considered [14-16].

Relying on the TCGA CRC data, we conducted a supervised analysis, integrating all of the multiple classes of available genomic feature data. The integrated data set included i) somatic mutations, ii) copy number alterations, iii) gene expression changes and iv) methylation. Our analysis uses elastic-net regression to estimate an optimal multiple linear regression of the clinical outcome on the space of genomic features. We analyzed this integrated genomic data set against clinical stage to delineate genes associated with advanced CRC.

Our study is unique and has specific strengths in many aspects compared to previous studies. Most importantly, with our integrated analysis method, we considered a full range of cancer genetic aberrations, otherwise described as genomic features. We identified specific cancer genes associated with advanced clinical stage; some of these genes have not been reported as being associated with cancer progression. The results of our analysis can be queried directly through a website (http://genomeportal.stanford.edu/tcga-crc).

## Methods

### TCGA CRC genomic data

Genomic data was obtained from the Broad Firehose (http://gdac.broadinstitute.org) which is one of the Genome Data Analysis (GDACs) for TCGA project. The data files from January 2013 analysis/standardization run of colorectal (COADREAD) cancer includes five genomics assays for each sample: DNA copy number variation, mRNA expression level by microarray/RNASeq, somatic mutations by whole exome sequencing, DNA methylation, and expression level of miRNA by RNASeq. Micro-RNA data was analyzed separately in our analysis because the frequency of missing data is relatively high and the general ambiguity in regards to identifying the specific gene targets subject to expression changes.

Clinical information of the samples was obtained from the Broad Firehose and UCSC cancer genome browser [17]. The availability of clinical parameter data for each sample was highly variable; therefore, we focused on those parameters that had the most complete annotation among the largest number of samples. We selected two major clinical parameters for elastic-net analysis: microsatellite instability (MSI), a molecular CRC feature associated with loss of DNA mismatch repair and clinical stage information. We also examined the individual variables of clinical stage via the TMN criteria: i) T is for extent of primary tumor invasiveness; ii) N derived from the number of cancer positive lymph nodes; iii) M is an indicator of metastasis in other organs beyond the primary site. These clinical outcomes were converted into ordinal values and used for subsequent elastic-net regression analysis (Additional file 1: Table S1).

We sought to differentiate between driver genetic aberrations in known and putative cancer genes versus passenger events in genes not related to cancer development. To eliminate non-contributing passengers, we relied on cancer genomic data resources cataloging known and putative cancer gene. These genes were identified from large-scale studies and curation of the scientific literature. We chose the genes for inclusion in our initial set using two data sources: Catalogue of Somatic Mutations in Cancer (COSMIC) and TCGA. We chose COSMIC because it has been both curated and validated. However, we were concerned that by imposing such high standards on genes that make it into the initial set, we might miss out on genes that are important, but not yet validated. To broaden the scope of our study and improved our identification of clinically relevant cancer genes, we added genes sets obtained from the TCGA project as part of their discovery studies.

Overall, the COSMIC database contains 484 genes that have been shown to be associated with cancer development and thus are established or candidate cancer genes [8]. The TCGA CRC analysis identified a large number of known and putative cancer genes including: 32 mutated genes, 353 genes with copy number changes, a 30 genes expression signature associated with tumor aggressiveness and a 344 genes methylation signature [13]. In addition, we included 20 genes known to be frequently mutated in colorectal cancer [18,19]. This resulted in a set of 1,192 known or putative cancer genes where there was significant genomic and literature information supporting their role in cancer (Additional file 2: Table S2).

### Data pre-processing and normalization

We conducted our analysis with Matlab (Mathworks, Natick, MA) and we provide the individual scripts used (Additional file 3). We used a multi-step procedure to update, normalize and filter the gene meta-data. First, we reviewed every gene symbol in all genomic data to the HUGO Gene Nomenclature (HGNC) [20]. According to the HGNC gene designation (March 2013), 55 gene symbols were not current and 15 genes were not protein coding genes. In total, 1,122 genes were in the candidate list after eliminating these 70 genes.

From the array data, we eliminated any features that had missing measurements for 3% or more samples. We imputed remaining missing measurements with the median across samples for each feature. For instance, there were 183 methylation array features lacked any values in 6 or more samples (≥ 3% of 197 samples) for clinical stage analysis. The imputation occurred with 26 features in methylation data and 6 features in mRNA microarray data.

Since mRNA levels were measured using both microarray and sequencing technology platforms, we combined these measurements for each gene using principal component analysis (PCA) [21]. PCA performs singular value decomposition on the probes by platform data matrix to yield factor weights for each platform. These weights were used in a linear combination to produce a single value for each gene,

$$\begin{array}{ll} \;\mathrm{mRNA}‒\mathrm{eigen}= & 0.8972\times \mathrm{mRNA}‒\mathrm{Array}+0.4417\\ & \times \mathrm{mRNA}‒\mathrm{Seq} \end{array}$$

where mRNA-array is the microarray measurement in log-fold change and RNA-Seq is the sequencing measurement in log(RPKM) for a particular gene. The mRNA-eigen value preserves on average 68.9% of the variance in the original data. Additional file 4: Figure S1) shows a scatter plot of mRNA measurements from both platforms and the principal eigenvector projection of the data.

We excluded CRC samples that were not measured in all genomic platforms. This filtration step retained 200 of the initial total 585 samples in the data set. Subsequently, we excluded the samples without clinical information (Additional file 5: Table S3). All 200 samples have clinical information about N status. However, there were several samples missing some clinical information; 3 samples missing clinical stage, 1 sample missing T status, 2 samples missing M status and 2 samples missing MSI status.

After pre-processing of data, we normalized the scale of each feature. Briefly, each genomic feature was normalized by the standard deviation of each gene’s measurement plus 10 percentile of the global standard deviations in each assay. This standard deviation correction factor is standard in microarray analysis [22] and minimizes the risk of generating outliers due to normalization. The normalization is,

$$\hat{g}\left(i,j\right)=\frac{g\left(i,j\right)}{\mathrm{sd}\left(g\left(i\right)\right)+sd_{10}\left(g\right)}$$

where g(i,j) is the value for feature i in sample j, sd(g(i)) is the standard deviation across samples for feature i, sd_10_(g) is the 10-percentile value of standard deviations across features and $\hat{g}\left(i,j\right)$ is the normalized feature value.

To prepare the final data set ready for analysis with the elastic-net, we simply combined the four genomic data sets (copy number, mutation, methylation, mRNA-eigen) into a single matrix representing 197 samples for clinical stage analysis. The data is available at our web portal (http://genomeportal.stanford.edu/tcga-crc/pages/datainformation).

### Elastic-net analysis

We used elastic-net regression to estimate an optimal multiple linear regression of the clinical outcome on the space of genomic features [23]**.** The elastic-net algorithm simultaneously performs linear regression to learn the coefficient weights associated with each genomic feature while limiting the number of predictors in the model to ensure the model is general. It performs well on independent data that was excluded from the analysis of the original primary data set. We used 10-fold cross-validation to identify the value of the regularization parameter that minimized the average mean-squared error on this held-out test set. An additional tuning parameter taking values between zero and one controls the inclusion of correlated predictors. The standard ‘Least Absolute Shrinkage and Selection Operator’ (LASSO), which is the prototype algorithm of elastic net, sets this value to zero to minimize the inclusion of correlated predictors; we set the value to the mid-point of 0.5. The resulting coefficients from the regularized regression were used to rank genes by their association with clinical features. It is important to note that the features identified by this procedure operate as a panel. While each individual feature may not predict the outcome well, in combination the prediction accuracy is improved as several other studies have shown this in various goal of using genomic data [24-29].

### Ranking scheme

In the primary results of elastic-net, a gene can appear at most four times when all four types of genomic data support the gene. The rank of each genomic feature is determined by its absolute value of regression coefficient in descending order. Our scoring scheme by ranks gives priority to genes in the top ranks or multiple ranks by two-step calculations. First, we calculated the unit score to weight the rank proportionally;

$$\mathrm{Unit}\;\mathrm{point}=1,000÷\sum_{r=1}^{n}r$$

where r is the rank and n is the total number of ranks in the result from elastic-net analysis. Second, the score of top ranked genomic feature will be n times higher than the score of the bottom ranked genomic feature;

$$\mathrm{Score}\;\mathrm{of}\;g\left(i\right)=\left(\mathrm{unit}\;\mathrm{point}\right)\times \left(n-r\left(i\right)+1\right)$$

where g(i) is the genomic feature, r(i) is the rank of the genomic feature of i, and n is the total number of ranks in the list. Finally, the score of a gene will be the sum of scores of genomic features from the gene. Therefore, a gene that has a highly ranked genomic feature and/or has multiple genomic features will have a higher score in overall.

### Assessment of profile robustness to variations in training data

Nonparametric bootstrap resampling was used to assess the robustness of the set of top ranked genes to changes in the training data as has been previously validated [30]. The complete data set was resampled with replacement 3,000 times and the elastic-net regression was recomputed for each bootstrap data set. Additional file 6: Table S4 details the count of the number of times a feature was selected by the elastic-net regression. Features that are consistently selected by the bootstrap regression have high rank and low variance in the rank are robust to variations in the training data set.

### Web implementation

To facilitate access to our analysis results, we provide a website that can be queried: http://genomeportal.stanford.edu/tcga-crc/. The TCGA COAD Resource runs on a 2x2.27GHz Quad Core Intel Xeon E5520 server, with 24GB memory, and Ubuntu 9.10 operating system. The web application is implemented in Ruby on Rails 4.0, running under Apache2 and Passenger 4. The underlying database is MySQL 5.1.7, which is hosted on a separate database server. Query and data download is via any current web browser. Recommended browsers and versions are: Chrome 28.0+, Internet Explorer 8.0+, Firefox 22.0+, Safari 5.0 + .

## Results

### Cancer genome atlas data for CRC

The TCGA CRC genomic data was obtained from the Broad Institute where the Tier 3 data (gene level calls that have been fully processed) are archived. Genomic data files from January 2013 analysis of colon and rectal cancer included five genomics assays for each sample: DNA copy number variation by single nucleotide polymorphism (SNP) array analysis, gene expression levels by microarray/RNASeq, somatic mutations by whole exome sequencing, DNA methylation via arrays and expression level of micro-RNA by RNASeq. These genomic data sets were integrated into a single data matrix for analysis (see Methods). Micro-RNA (miRNA) data was analyzed separately because the frequency of missing data is relatively high and the general ambiguity in regards to identifying the specific gene targets subject to expression changes.

**Figure 1:**
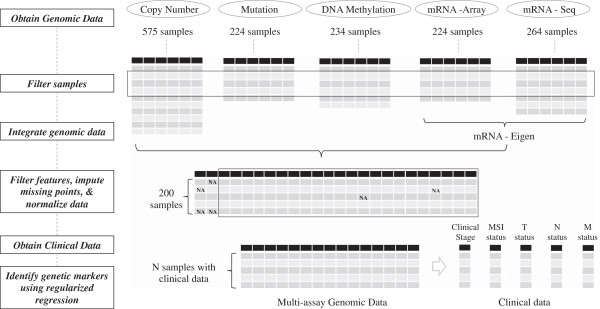
**Overview of the data analysis process.** Genomic data was collected from TCGA and Broad FireHose websites. Samples have complete data for mutations, copy number alteration, gene expression and methylation. Gene expression from microarrays and RNASeq were combined using principal component analysis into a single measure for each gene and then concatenated to the data for the other assays. Missing data was imputed using the median value across samples. Finally, regularized regression (elastic-net) was used to identify a minimal set of features that delineated clinical stage, the extent of tumor invasion into the colon, metastasis in lymph nodes, metastasis in other organs and microsatellite instability (MSI).

We used a gene-centric approach for this study. For each gene, there were four genomic features represented by i) mutations, ii) copy number variation (CNV), iii) gene expression and iv) methylation status. We selected a candidate set of 1,185 known or putative cancer genes for our analysis. Using this approach i) reduces the issue of passenger events of no genetic significance, ii) provides an improved regression analysis and iii) facilitates interpretation of results according to their potential role in biological processes related to cancer. The genomic data of the selected genes was downloaded, pre-processed and integrated into a single data matrix for elastic-net analysis (Figure 1). As a normalization step to facilitate our study, we rescaled the features by their standard deviation across samples. This step improved the robustness of the analysis. This single combined matrix has genomic values of genes (independent variables) and the clinical table contains specific clinical parameters such as microsatellite instability (MSI) status and clinical stage.

### Application of elastic net regression to integrated genomic features

We used elastic-net regression to estimate an optimal multiple linear regression of the clinical outcome on the integrated data set of all genomic features [23]**.** The elastic-net algorithm performs linear regression to learn the coefficient weights associated with genomic features simultaneously. Each coefficient in the elastic-net regression measures the partial correlation between the predictor feature (e.g. CNV, gene expression, methylation level or mutation status) and the outcome (e.g. clinical stage).

**Figure 2:**
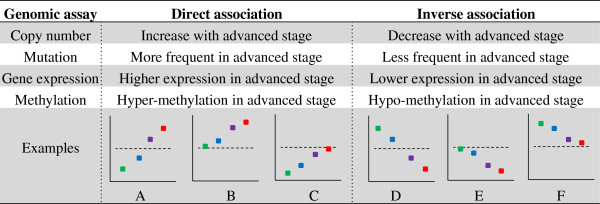
**The interpretation of elastic net association for the comparison of low versus advanced clinical stage colorectal cancer.** This figure shows comparison of lower versus higher advanced stage in direct/inverse association. The Examples row shows clinical stage I (green), II (blue), III (purple) and IV (red) respectively. The x axis represents clinical stage while y axis represents genomic changes. Dotted lines imply normal status.

In general, a partial correlation is a measure of association between two variables after controlling for other variables – in this case the other variables are the other predictors in the regression (Figure 2). A positive coefficient – “direct association” - indicates that as the level of the predictor increases, the outcome increases after controlling for all other significant features. We indicate a direct association as a “**↑**”. Likewise, a negative coefficient – “inverse association” - means that as the predictor decreases, the outcome increases after controlling for other predictors (Figure 2). We indicate an inverse association as a “↓”.

Figure 2 shows comparison of lower versus higher advanced stage in direct/inverse association. The individual dots in each Case graph (Examples) represent stage I, II, III and IV respectively in order. The X axis represents clinical stages I through IV while Y axis represents genomic changes. Dotted lines imply normal status. Extra attention is required for Cases C and F. Citing an example, Case C denotes that a reduced copy number such as might occur with a genomic deletion is more frequently observed in early stage compared to advanced stage CRC. Citing the reverse scenario, in Case F the amplification and thus increased gene copy number is observed in early stage while no copy number changes are noted in advanced stage CRC.

The resulting coefficients from the regularized regression were used to rank genomic features by their association with clinical features. The score of the genetic features was determined proportionally to their ranks and the score of each gene is the sum of all scores of its selected features (see Methods). This scoring scheme prioritizes genes that are supported by multiple genomic features and/or a highly ranked genomic feature. It is important to note that the features identified by this procedure operate as a panel. Several other genomic analysis studies have demonstrated that while an individual genomic feature may not predict the outcome, combining multiple genomic features improves the prediction accuracy [24-29].

### The performance of elastic-net

To evaluate the utility and performance of elastic-net regularized regression for integrative genomic analysis, we examined two aspects of its performance: i) selection of genes that are known to be associated with a clinical phenotype and ii) combination of heterogeneous genomic data from different assays. First, we applied elastic-net on a data set in which the true features and clinical associations are known. We generated four additional synthetic genes and their corresponding genomic features that are associated with clinical stage; values were generated for copy number alterations, mutation, methylation, and gene expression that were primarily present in stage III and IV CRC (Additional file 7: Figure S2). These synthetic data were appended to the fully integrated CRC data matrix. In the case of the mutation feature, we increased the frequency of mutation in each stage incrementally. Our elastic net analysis correctly identified all four synthetic genomic features and their association with clinical stages of CRC as top candidates.

We applied elastic-net regularized regression method to a TCGA study of glioblastoma (GBM) [31]. Using the same reported mutation and clinical outcome data used in the original publication reporting the TCGA GBM data, we identified *IDH1* to be associated with the days to death outcome. The TCGA GBM study did not identify mutations in *IDH1* as being clinically relevant but more recently, other studies have identified them to be indicators of poor prognosis [32].

Second, we examined the effects of variable value scales for any genomic feature occurring with the integrated CRC heterogeneous genomic data. On the integrated genomic data matrix, we increased each value 100 fold and determined any variation in the final results after elastic-net analysis. This multiplication of assay values transformed the scale of each genomic feature. Elastic-net analysis was run on this modified data set. The list of selected genes was not significantly altered.

### Genomic features and genes associated with MSI

MSI is a molecular phenotype associated with loss of DNA mismatch repair function. In MSI-positive CRCs, there is a substantial increase in mutations occurring in microsatellite sequences within the coding region of critical cancer genes. The genetic basis of MSI-positive CRCs has been extensively studied and the identification of MSI mutations has revealed critical cancer genes. We conducted a supervised analysis with MSI status. We determined whether our approach could identify the important cancer genes involved in MSI-positive CRC that are already known from prior studies.

Elastic-net analysis of mutation data with MSI status identified 21 genes including *TGFBR2*, *CASP8*, and *ACVR2A* while the analysis of methylation data listed 30 genes such as *MLH1*, *FLVCR2*, and *EFNA1* (Table 1). The *TGFBR2* gene encodes a receptor for the TGF-β pathway and is a known cancer driver gene in MSI-positive CRC. This gene has a homopolymer (A)_10_ tract in exon 4 which is mutation hotspot in MSI-positive CRC. This particular deletion markedly reduces mRNA levels, presumably due to nonsense-mediated decay [33]. The silencing of *MLH1* by hypermethylation results in the loss of DNA mismatch repair (MMR) activity and methylation of *MLH1* is frequently observed in sporadic MSI-positive CRC [34]. Our integrative analysis identified the methylation of *MLH1* as the top ranked MSI-associated candidate and the mutation of *TGFBR2* as 6th top candidate among a total of 64 selected features (Table 1). In addition, the 9th ranked genomic feature was lower gene expression of *MLH1* as one would expect in the context of *MLH1* hypermethylation. According to TCGA analysis (January 2013), their coefficient between methylation and mRNA expression was -0.556 with p value of 4.43E-04. We also identified that a lack of copy number alterations in *SMAD4* associated with MSI. This inverse relationship is consistent with the fact that MSI-positive mutations demonstrate a significantly lower level of copy number alterations affecting cancer genes. Overall, our results demonstrate the success of our elastic-net based analysis in identifying the MSI-associated genes. Additional file 8: Table S5 lists the full list of identified genes associated with MSI status using separate data sets.

**Table 1:** Top 10 genes associated with MSI

	**Mutation alone**		**Methylation alone**		**Integrated data**	
**Rank**	**Gene**	**Sign**	**Gene**	**Sign**	**Gene**	**Sign**
1	*TGFBR2*	↑	*MLH1*	↑	*MLH1 (methyl)*	↑
2	*CASP8*	↑	*FLVCR2*	↑	*PDE6B (mut)*	↑
3	*ACVR2A*	↑	*EFNA1*	↓	*POLR1D (mut)*	↑
4	*MSH3*	↑	*CDK12*	↑	*FLVCR2 (methyl)*	↑
5	*MAP3K6*	↑	*HOXC11*	↓	*DAK (mRNA)*	↑
6	*GATA2*	↑	*ZNF318*	↑	*TGFBR2 (mut)*	↑
7	*MACF1*	↑	*SEPT5*	↑	*SEPT5 (methyl)*	↑
8	*BRAF*	↑	*MIER3*	↑	*EFNA1 (methyl)*	↓
9	*GPHN*	↑	*ELK4*	↓	*MLH1 (mRNA)*	↓
10	*MAEA*	↑	*SDHD*	↓	*STK32B (mut)*	↑

Gene ranking is based on the regression coefficient (see Methods). Genomic assay and associated feature is indicated in parentheses for integrative analysis of the combined genomic data. The symbol ↑ indicates increased frequency (mutation) or level (methylation) in MSI. The symbol ↓ indicates decreased frequency (mutation) or level (methylation, gene expression) in MSI.

### Identification of genes associated with advanced clinical with integrated analysis

**Figure 3:**
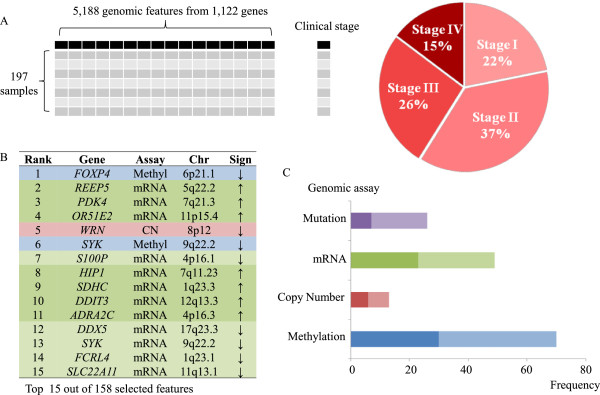
**Model for prediction of clinical stage using regularized regression. (A)** We used an integrative matrix of 5,188 genomic features from 1,122 genes in order to predict clinical stage using elastic net regularized regression. **(B)** The top 15 features ranked by coefficient in the model are shown. **(C)** Methylation features dominate the predictor set, but gene expression, copy number alteration and mutation features also play a critical role. The darker color indicates the direct association while the lighter color represents the inverse association.

As shown in Figure 3A, the elastic-net algorithm identified genes across the different platforms that showed and association with clinical stage (e.g. Stage I, II, III, and IV). As shown in Figure 3B, 158 genomic features were selected for their association with stage III and IV CRC. We determined the ranks of these genes based on their absolute value of regression coefficient at the minimum mean squared error (MSE) determined by 10-fold cross validation. The data and analysis results are available at open access website (http://genomeportal.stanford.edu/tcga-crc).

Overall, 158 genomic features associated with 143 genes delineated advanced clinical stage CRC. Fifteen genes demonstrate multiple genomic features (Additional file 9: Table S6). Methylation of the gene *FOXP4* was the top ranked genomic feature that has the largest absolute value of regression coefficient. Since *FOXP4* has a negative coefficient, it implies that *FOXP4* is less methylated in advanced CRC. Only one gene, *WRN*, had three selected genomic features (copy number, mRNA, and methylation) while 13 genes had two genomic features. Other top ranked signatures such as *REEP5*, *PDK4* and *OR51E2* show higher gene expression in advanced stage CRC.

Among the 158 genomics features associated with advanced clinical stage, 45.5% are methylation, 16.8% are mutations, 31.8% are gene expression related and 8.4% are copy number alterations (Figure 3C). Gene expression features are the most common among the top 15 ranked features (12 out of top 15). An inverse association (58.2%) is more common than direct association (41.8%) although copy number and gene expression had similar frequency between inverse and direct association. For example, a higher portion of inverse association in methylation indicates hypermethylation in advanced stage; as a result more genes will have lower expression in advanced stage disease.

### Identification of genes associated with advanced clinical stage using individual genomic feature data sets

**Figure 4:**
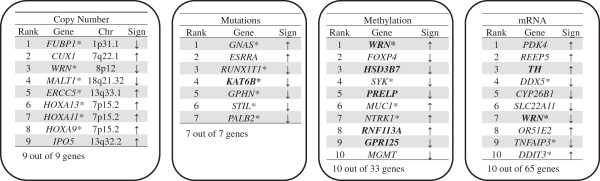
**Prediction of clinical stage from individual genomic assay data sets.** Using individual data from separate genomic assays, we conducted a supervised analysis with clinical stage. Some of these results agree with previous analysis by the TCGA; all of the features shown in bold to be associated significantly (p value < 0.01) with clinical stage according to TCGA study. All association signs are identical between our analysis and TCGA analysis. A star symbol indicates cancer genes annotated by COSMIC.

We ran the same elastic net regression analysis using individual genomic feature data sets (e.g. mutations alone, copy number alterations alone, etc.) compared to clinical stage (Figure 4). Elastic-net identified genomic features associated with advanced clinical stage CRC: this includes nine copy number aberrations, seven mutations, 65 gene expression changes, and 33 methylation events affecting specific genes (Additional file 9: Table S6). Only eight genes were identified with multiple genomic features: *WRN, SYK, MGMT, CAPSL, ADRA2C, GNAS, IOP5* and *SEMA3B*. The other 96 genes were associated with only an individual genomic assay.

Our results overlapped with some of the findings of the recently published TCGA CRC study (Figure 4). The TCGA study used each genomic data set separately (e.g. mutations alone, etc.) without considering the behavior of other genetic aberration features. For their study, they determined the association between specific genetic aberrations with a number of clinical parameters including clinical stage by Fisher’s test (p < 0.01). Fifteen methylation features (out of 33), 7 gene expression features (out of 65), and 1 mutation feature (out of 7) in our study were shown to have the significant and overlapping association with clinical stage in TCGA study. Unlike our study, the original TCGA analysis of copy number alterations list large genomic intervals with multiple genes and did not identify specific genes associated with clinical stage.

We observed several differences between the integrated data set incorporating all genomic features versus an analysis of individual genomic feature data set (e.g. mutations alone, copy number alone, etc.). The integrative analysis had a better predictive power based on the mean squared error (Table 2). Likewise, different genomic features were selected from both approaches. Seventy-seven out of 142 genes from integrative analysis were not listed in any separate assay analysis. This indicates these features are predictive only in the context of analyzing other genomic features representing different classes of genetic aberrations. However, the top ranked genes were quite consistent between two approaches. Seventeen of the 20 genes in integrative analysis were also identified in our analysis of the individual data sets. From the analysis of individual genomic platform data sets, the relative rankings of genes also changed compared to the fully integrated analysis. For example, when analyzing only the methylation data, the top ranked gene, *WRN*, dropped to a rank of 8th while the 2nd highest ranked gene, *FOXP4*, was the top ranked gene.

**Table 2:** The mean squared errors (MSE) of 10 fold cross validation from Elastic-net of genomics data against clinical stages of CRC

**Genomics**	**MSE**
Integrative genomics	0.78224
Copy Number alone	0.96308
Mutation alone	0.97295
Methylation alone	0.91856
Gene expression alone	0.79906

Integrative analysis - the combined genomic data – showed the least MSE compared to separate genomics analysis. Therefore, the improvement was observed in integration of genomics data.

### Candidate genes delineating advanced CRC ranked by multi-genomic feature score

We used our scoring scheme to prioritize the candidates (see Methods). As we noted previously, the integrative genomic analysis obtained total of 142 unique genes (Table 3, Additional file 9: Table S6) with 158 genomic features indicative of different genetic aberrations. In terms of their annotation, 56 genes are annotated in COSMIC cancer genes, two genes are MSI targets and 95 genes are reported as significant in the TCGA CRC study. Thirteen genes were listed as cancer genes in both COSMIC and the TCGA CRC study. Fourteen genes had genomic features from more than one assay. Generally, the top ten ranked genes all had multiple genomic features associated with advanced clinical stage.

**Table 3:** Top 25 candidates associated with advanced clinical stage

	**Gene**		**Elastic-net Feature Rank**			
**Gene**	**Chr**	**Score**	**Copy Number**	**Gene Expression**	**Methylation**	**Mutation**
WRN*	8p12	27.03	5 ↓	106 ↓	29 ↑	
SYK*	9q22.2	24.09		13 ↓	6 ↓	
DDX5*	17q23.3	18.94	70 ↓	12 ↓		
*ADRA2C*	4p16.3	18.13		11 ↑	81 ↓	
GNAS*	20q13.32	16.9			39 ↓	68 ↑
*SEMA3B*	3p21.31	16.66		82 ↓	28 ↓	
*HSD17B2*	16q23.3	14.45	17 ↑	120 ↑		
*TTN*	2q31.2	13.72		80 ↓		66 ↓
FHIT*	3p14.2	13.56			71 ↑	77 ↓
HIST1H4I*	6p22.1	12.82		35 ↑	122 ↓	
*FOXP4*	6p21.1	12.74			1 ↓	
*REEP5*	5q22.2	12.66		2 ↑		
*PDK4*	7q21.3	12.58		3 ↑		
*OR51E2*	11p15.4	12.49		4 ↑		
*S100P*	4p16.1	12.25		7 ↓		
HIP1*	7q11.23	12.17		8 ↑		
*ZNF570*	19q13.12	12.09		50 ↑		116 ↓
SDHC*	1q23.3	12.09		9 ↑		
DDIT3*	12q13.3	12		10 ↑		
CRTC1*	19p13.11	11.92			130 ↑	38 ↓
FCRL4*	1q23.1	11.84		14 ↓	155 ↓	
*SLC22A11*	11q13.1	11.6		15 ↓		
*FLT1*	13q12.2	11.51		16 ↑		
*CYP26B1*	2p13.2	11.35		18 ↓		
*RNF113A*	Xq24	11.27			19 ↑	

We ranked the genes by selected feature scoring (see Method for scoring scheme). This scoring scheme selects genes that are supported by multiple and/or highly ranked genomic features. Elastic-net feature rank indicates the rank of individual assay features, where ↑ indicates a direct association with the feature value and ↓ indicates inverse association with the feature value. COSMIC-annotated cancer genes are marked by *.

**Figure 5:**
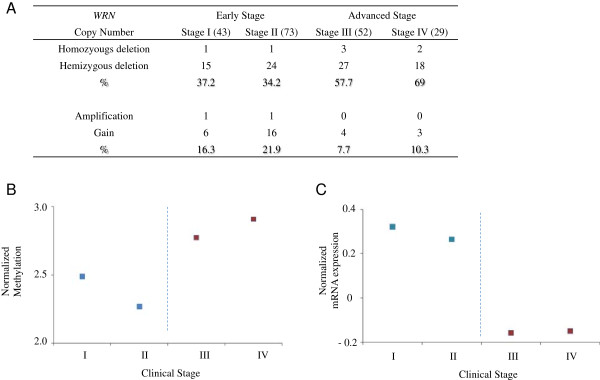
***WRN *genomic variations are associated with clinical stage.***WRN* copy number alterations **(A)**, methylation **(B)** and gene expression **(C)** show differential levels between stage I & II disease and stage III and IV disease. The deletion of *WRN* by copy number alterations is higher at ~56% in stage III & IV than in stage I & II (~34%). Methylation is slightly higher and gene expression is generally lower in advanced clinical stage disease.

*WRN* was the top ranked gene associated with advanced clinical stage CRC; *WRN* was implicated by multiple genomic features - copy number alterations, methylation, and gene expression. The *WRN* gene demonstrates hyper methylation (↑), lower copy number (↓) and decreased mRNA expression (↓) in stages III and IV (Figure 5). Overall, these different genomic features suggest that the expression of *WRN* is decreased in advanced clinical stage CRC. To determine the effect of other genes in proximity to *WRN* at locus 8p12, we appended the genomic features from adjacent genes neighboring *WRN* to the integrated data matrix. This included 13 genes telomeric and 6 genes centromeric to the 8p12 locus of *WRN*. Afterwards, we conducted elastic-net analysis of the original matrix. The analysis continued to identify *WRN* as the leading candidate gene even when taking into account the adjacent genes. The frequent deletion and methylation of WRN in advanced/CpG island methylation phenotype (CIMP) CRC has been reported [35-37]. This is external data supporting *WRN’s* potential role in CRC.

*SYK* had the second highest score by mRNA expression and methylation feature. Given the central role of *SYK* in transferring activated immunoreceptors within B-cells, lower *SYK* gene expression may be involved in dampening the immune response against cancer cells. Furthermore, this gene may have a potential role in the development of human breast carcinomas [38].

We assessed the robustness of the top ranked candidates list by nonparametric bootstrap analysis (see Methods). With a 3,000 bootstrap resampling, we found that the top 25 genomic features from our initial analysis were selected repeatedly from the complete integrated genomic data set (Additional files 6: Table S4). For example, elastic-net analysis of a 3,000 bootstrap resampling data set identified gene expression of *PDK* 2,361 times (78.7% among 3,000 bootstrap analysis). The *WRN* copy number and methylation features were identified 2,097 times (69.9%) and 1,710 time (57.0%) respectively. When we conducted an independent elastic net analysis to the separate individual platform genomic data sets with 3,000 bootstrap resampling, *WRN* was again frequently identified; 2,718 times (90.6%) in copy number alone, 2,511 times (83.7%) in methylation alone, and 1,749 times (58.3%) in mRNA expression alone.

We examined the characteristics of multiple genomic features among the 14 top ranked genes associated with advanced stage clinical disease. The combination of gene expression and methylation implicate seven genes; this is the most frequent combination of features that came from our analysis. Three genes were selected with both gene expression and copy number. This suggests that methylation has a more significant influence on gene expression compared to copy number changes. For example, two genes have hypo-methylation and higher gene expression. Copy number changes are directly associated with gene expression in all three cases. In addition, we also observed the combination of copy number – methylation (1 case), mutation – methylation (3 cases), and gene expression – mutation (2 cases).

### Genomic features associated with individual parameters of clinical stage

**Figure 6:**
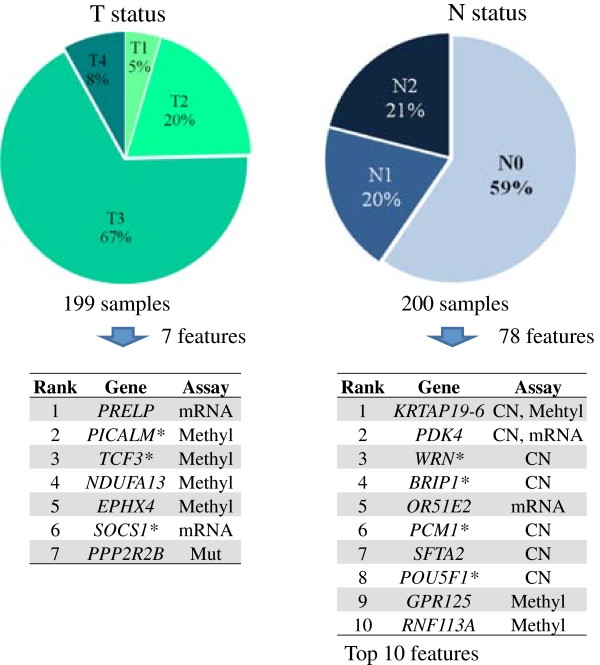
**Genes delineating T and N status.** We conducted elastic-net analysis with the individual parameters of TNM clinical stage criteria. The associated number of genes included 7 for T status and 78 for N status, but no gene for M status.

We conducted a separate supervised analysis using the individual parameters of TNM clinical stage criteria (Figure 6). As noted, T is assigned by extent of tumor invasion, N represents regional lymph node cancer involvement and M represents the presence of metastatic cancer in other organs. Elastic-net identifies seven genomic features associated with higher level T status while 78 genomic features including ones that affected *WRN* are associated with N status (Additional file 10: Table S7). Interestingly, the three top-ranked candidates (*WRN*, S*YK* and *DDX5*) based on our supervised analysis of clinical stage are also associated with N status. This result indicates that lymph node metastasis is critical to advanced stage compared to T status, which is expected by AJCC staging method. There are no genes that delineated M status independently. The smaller number of stage IV samples (15%) may have affected our sensitivity for identifying genes associated with distant metastasis in other organs.

**Figure 7:**
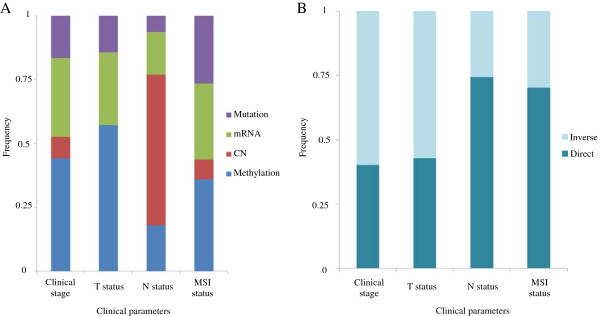
**Predictive value of genomic feature varies by clinical parameter being predicted. (A)** Each clinical parameter has different dominant genomic feature. Methylation features dominate the predictor list for clinical stage and T status while copy number alterations are most frequent for predicting N status. **(B)** The distribution of associate direction varies among clinical parameters.

Each clinical parameter has its own unique distribution of genomic features (Figure 7A). When considering lymph node status (e.g. N), copy number alterations were most common genomic feature identified while methylation features were most common in the prediction of tumor invasiveness (e.g. T). As one may expect, mutations were relatively common feature for MSI status compared to other clinical parameters. Inverse associations are dominant in tumor invasiveness (T) while direct associations were predominant in lymph node (N) status and MSI (Figure 7B). The frequent direct association in N status and MSI implies that the gain or higher expression genetic aberrations occur more frequently in advanced versus early clinical stage. Dominant inverse association in T status and clinical stage suggests that loss of or lower expression of genes is a more frequent in advanced stage clinical disease. However, all 25 genomic features common in clinical stage and N status had identical association directions; 14 with direct associations and 11 with inverse associations.

If one includes the results from all of the tested clinical parameters, a total of 237 genes are identified. There are no genes selected independently that predicted all four clinical parameters. The majority of genes (80.5%) are associated with a single clinical parameter. Three genes were identified repeatedly among 3 different clinical parameters: *BCL2*, *TRERF1*, and *MGMT*. Forty-three genes were associated with two different clinical parameters.

### Identification of miRNAs associated with CRC clinical stage

As noted previously miRNA data was analyzed separately because of extensive gaps in the data and the general ambiguity about which genes are regulated by miRNAs. We ran an elastic net regression on miRNA data separately in comparison with clinical stage. We used the same 197 CRC samples as the integrative genomic analysis. There are two types of genomics data for miRNA; copy number alterations and expression. The copy number data included 2,260 miRNAs while the expression data covered 420 miRNAs. First, we integrated both miRNA copy number and expression into a single matrix. However, this combined data analysis did not produce any candidates associated with advanced clinical stage. Second, we ran the elastic net regression separately on copy number and expression data. From the expression data set, our analysis identifies 33 miRNAs associated with clinical stage (Additional file 11: Table S8). The top candidate, *MIR21*, was suggested as a potential diagnostic marker of colorectal cancer [39] and involved in tumor growth in breast cancer [40]. Interestingly, *MIR21* is located at 17q23.1 in relative proximity to the top 3rd stage associated gene, *DDX3* (17q23.3). None of 33 miRNAs are located in loci for the other top candidate genes such as 8p12 (*WRN*), 9q22 (*SYK*), or 4p16 (*ADRA2C)*.

## Discussion

We conducted a regularized elastic-net regression analysis of heterogeneous genomic data that encompassed multiple classes of genetic aberrations (i.e. genomic features) in colorectal cancer. We identified 158 genomic features associated with advanced clinical stage with 10-fold cross validation (Figure 3) and ranked these genes based on by their regression coefficient and level of support from multiple assay modalities (Table 3). Our integrative analysis approach provides a better picture about the distribution of genomic features associated with clinical parameters, which cannot be obtained by separately analyzing individual classes of genetic aberrations such as mutations alone (Figure 7). For example, our integrative analysis identified specific mutations that delineate clinical stage in the context of overlapping genomic features. This was not possible when analyzing mutations alone without the genomic context of other genetic aberrations. We also demonstrated that the predictive power was relatively improved using our integrative analysis approach (Table 2) as several other studies have demonstrated previously [24-29]. Despite of slight increase in predictive power, integrative analysis enabled us to identify critical genes that are supported by more than one genomic biomarker. Several top candidates such as *GNAS* (supported by methylation and mutation) and *FHIT* (supported by methylation and mutation) would not be detected if analysis was solely based on gene expression data.

The leading candidate, *WRN*, showed the higher number of genomic features that can delineate advanced clinical stage CRC (Figure 5). *WRN* is a known tumor suppressor. Hereditary germ line mutations in the *WRN* gene cause the adult-progerioid genetic disorder known as Werner’s syndrome (WS). This disorder is associated with an increased risk of cancer [41-43]. The increased risk of cancer in individuals with WS has established the role of *WRN* as a tumor suppressor gene. However, genetic aberrations in *WRN* have never been described in sporadic CRC development. *WRN* is a human RecQ DNA helicase family and has both the characteristic 3′ to 5′ helicase activity and also a 3′ to 5′ exonuclease activity. Generally, we observed the *WRN* was subject to hemizygous gene loss, a category of genetic aberrations that are increasingly being scrutinized for a specific role in cancer development. Solimini et al. recently provided evidence that the gene dosage changes as result of hemizygous deletions play an important role in tumor progression [44]. We are pursuing translational validation studies and further experiments to address the role of *WRN* in advanced CRC.

The results from analysis of the individual clinical parameters of the TMN criteria provided us with additional biological insight. There was significant overlap between genes associated with advanced clinical stage and N status, more so than any other comparison among the various clinical parameters we examined. This may be an indication that genes associated with lymph node invasion are relatively critical for delineating advanced stage of CRC compared to other parameters such as tumor invasiveness level. This conclusion is supported by the fact that lymph node status remains the most important and reproducible prognostic clinical parameter for colon cancer [45]. Interestingly, we did not identify a gene specific for distant organ metastasis. This may be related to the smaller number of stage IV CRCs in early releases of the TCGA data set or the potential greater role of lymph node metastasis as an early indicator of metastatic progression. By grouping stage III and IV patients as advanced disease, we improve the sample size at the risk of increasing within-group heterogeneity. As the TCGA CRC data set matures, we will have opportunity to analyze a large data set for associations.

We evaluated the performance of elastic-net on the large genomic data set with clinical information by using control data sets. Our analysis successfully identified the clinically relevant genomic signatures in three cases: i) Identification of synthetic genes that have stage-associated genomic features, ii) Identification of the *IDH1* gene from GBM mutation data and iii) identification of methylation on *MLH1* and mutations on *TGFBR2* for MSI status of CRC. In the case of the CRC stage association, some of the selected genomic features for CRC stage from each genomic assay were consistent with the results from the TCGA CRC study (Figure 4). Our study supports the overall reliability of the candidates associated with clinical stage from elastic-net.

Interestingly, cancer genes (e.g. *TP53*, *KRAS*, *APC*) with the highest frequency of genetic aberrations were not among the genes identified in delineating advanced clinical stage of CRC. These cancer driver genes were mutated frequently across all clinical stages. For instance, *APC* was mutated in 90%, 74%, 77%, and 93% of stage I, II, III and IV respectively. Therefore, our interpretation is that while these cancer drivers play a critical role in the initial neoplastic development and maintenance, they play lesser role for influencing metastatic progression and advanced clinical stage.

Our study is distinct and novel compared to the TCGA CRC study; unlike TCGA, we considered four major features of genetic aberrations simultaneously rather than specific genetic aberrations in isolation. As we demonstrate, this improves the performance of our approach; it leads us to some gene candidates delineating advanced clinical stage not previously recognized. Integration of different genomic data gives us more informative results as we have shown it in this study. Multiple genomic features enable us to prioritize the gene aberration for follow-up validation studies. Another feature of our study is the flexibility of our analysis approach. Using elastic net, we can readily modify our bioinformatics pipeline to consider other clinical parameters. In the future, we anticipate applying additional clinical parameters such as drug response for our analysis as these data become available.

One of the main limitations of our study is the absence of an independent data set for validation. We demonstrated the robustness of our candidates by bootstrap resampling. We also considered over 11 other genomic studies of CRC that looked at differences among early versus advanced stage disease [46]. Only two analyzed tumors from all clinical stages usually with less than 80 samples. In addition, all of these studies used older gene expression microarrays lacking the number of features see in the TCGA and thus never reached the level of comprehensive multiple-platform analysis or high genomic resolution as was conducted by the TCGA. For this analysis, the ability to integrate heterogeneous genomic features was critical and the limitations of the other data sets made them less useful for our integrated analysis. As a validation analysis with a single platform, we opted to use an independent set of 354 samples with both clinical data and copy number variation data from the expanded TCGA CRC data. This separate analysis validated *MALT1* as a top hit when only considering copy number analysis.

We also considered an integrative analysis of independent CRC samples from TCGA. At the time of this study, many of the TCGA samples have not undergone analysis with all of the genomic assays. Many of the other CRC samples, outside of the ones we use, lack sufficient clinical data. In the future, with the completion of the TCGA study and adequate clinical annotation afterwards, we will use the additional data sets from independent samples from our original analysis.

To improve our analysis, we are testing methods to integrate disparate classes of data; for example, this genomic data covers a range including binary for mutation, categorical for copy number, and continuous for mRNA and methylation. One potential approach involves turning the values of every genomic assay into categorical variable by discretization. For example, GISTIC is a method for producing discrete values for discriminating significant copy number alterations. However, discretization is less applicable to continuous data sets such as gene expression and methylation. Another possibility involves converting every genomic assay feature into a continuous value. For example, we can use several programs that predict the functional impacts of mutations, thus convert binary mutation values (e.g. stop mutation, substitutions, etc.) into continuous pathogenicity values. We will test whether homogeneity in different genomic values may improve the predictive power of our analysis and improve the ranking of genes in terms of their importance biologically and clinically.

## Conclusions

Leveraging the expansive and comprehensive data sets of the TCGA, we developed a robust and straightforward approach to integrate and analyze heterogeneous cancer genomic data sets. Based on a supervised comparison of clinical parameters indicative of advanced CRC, we utilized in parallel mutation, gene expression, copy number alteration and methylation data and we identify genes associated with advanced clinical stage, several of which have not been identified previously.

## Abbreviations

CRC: Colorectal cancer; TCGA: The cancer genome atlas; SNP: Single nucleotide polymorphism; TGF-β: Transforming growth factor β; miRNA: micro RNA; MSI: Microsatellite instability; GBM: Glioblastoma; MMR: Mismatch repair; MSE: Mean squared error; GDACs: Genome data analysis centers; HGNC: HUGO gene nomenclature; PCA: Principal component analysis; LASSO: Least absolute shrinkage and selection operator; WS: Werner’s syndrome; COSMIC: The Catalogue of somatic mutations in cancer; RPKM: Reads per kilo base per million; T status: Extent of tumor invasion; N status: Regional lymph node cancer involvement; M status: Presence of metastatic cancer in other organs; CIMP: CpG island methylation phenotype.

## Competing interests

The authors declare that they have no competing interests.

## Authors’ contributions

PF, HJL and HPJ designed the study. HJL and PF conducted the analysis. HPJ supervised and coordinated the overall study. All authors revised, read and approved the final manuscript.

## Pre-publication history

The pre-publication history for this paper can be accessed here:

http://www.biomedcentral.com/1755-8794/6/54/prepub

## Supplementary Material

Additional file 1: Table S1 Change clinical parameters into ordinal values. Click here for file

Additional file 2: Table S2 The list of selected genes. Click here for file

Additional file 3 Analysis scripts and commands. Click here for file

Additional file 4: Figure S1 Gene expression eigen scatter plot. Click here for file

Additional file 5: Table S3 The list of samples with their clinical information. Click here for file

Additional file 6: Table S4 Results of bootstrap analysis. Click here for file

Additional file 7: Figure S2 Elastic-net analysis of synthetic genetic features. Click here for file

Additional file 8: Table S5 The list of genes associated with MSI. Click here for file

Additional file 9: Table S6 The list of genes associated with clinical stage. Click here for file

Additional file 10: Table S7 The list of genes associated with T status and N status. Click here for file

Additional file 11: Table S8 The list of micro RNAs associated with clinical stage. Click here for file
