# Supplementary figures and images for: Systematic genomic identification of colorectal cancer genes delineating advanced from early clinical stage and metastasis
Source: BMC Med Genomics. 2013 Dec 5;6:54. doi: 10.1186/1755-8794-6-54 (PMC3907018; doi:10.1186/1755-8794-6-54)

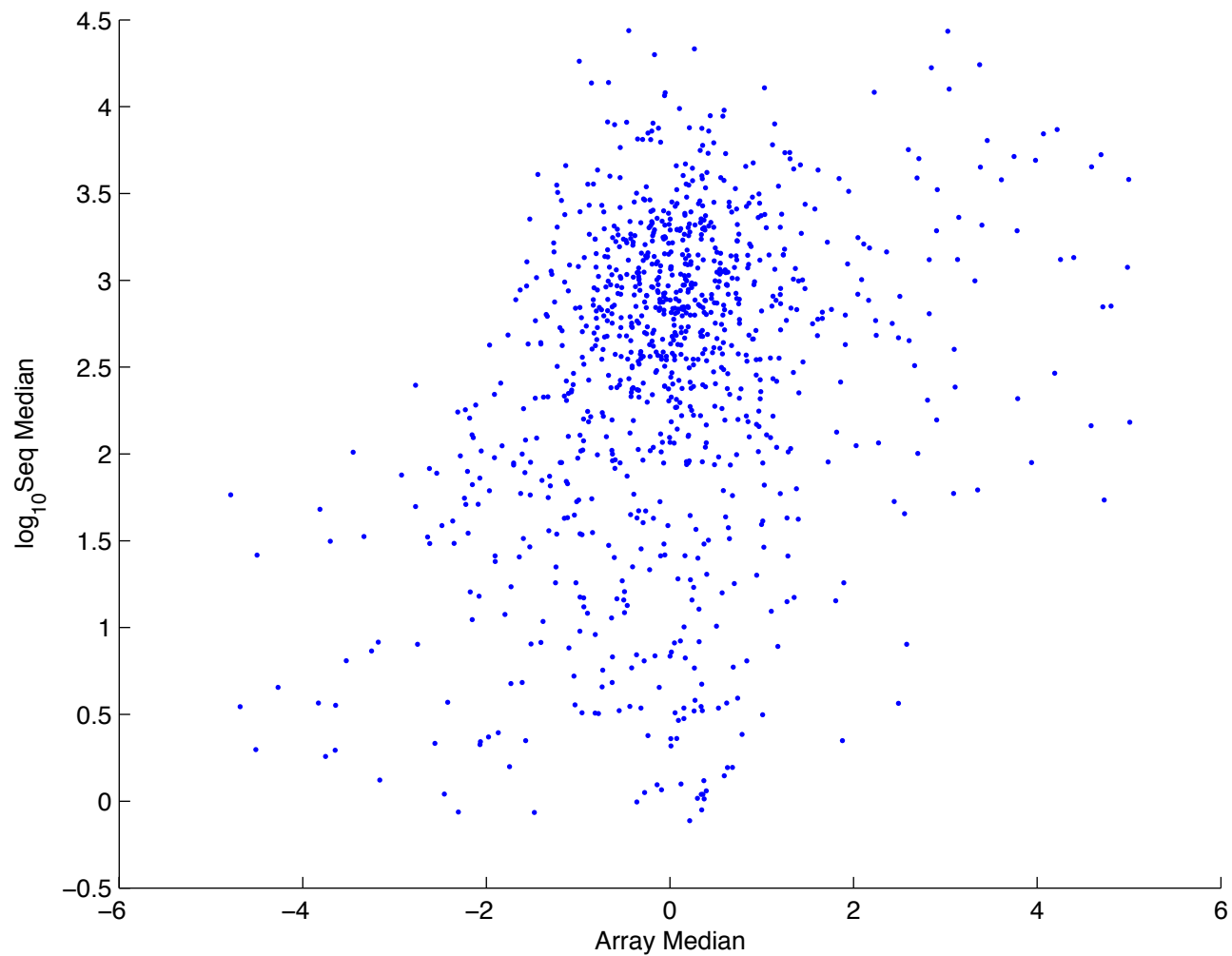

Supplement: Additional file 4: Figure S1 — Gene expression eigen scatter plot. [file 1755-8794-6-54-S4.pdf]
