# Supplementary material for: Systematic genomic identification of colorectal cancer genes delineating advanced from early clinical stage and metastasis
Source: BMC Med Genomics. 2013 Dec 5;6:54. doi: 10.1186/1755-8794-6-54 (PMC3907018; doi:10.1186/1755-8794-6-54)
Supplement: Additional file 7: Figure S2 — Elastic-net analysis of synthetic genetic features. [file 1755-8794-6-54-S7.pdf]

Stage	*Synthetic A* Copy Number	*Synthetic B* Copy Number	*Synthetic C* Mutation	*Synthetic D* mRNA	*Synthetic E* Methyl
Stage I	-2	0	25%	0.5	0.25
Stage II	-1	1	50%	1	0.5
Stage III	1	2	75%	1.5	0.75
Stage IV	2	2	100%	2	0.95

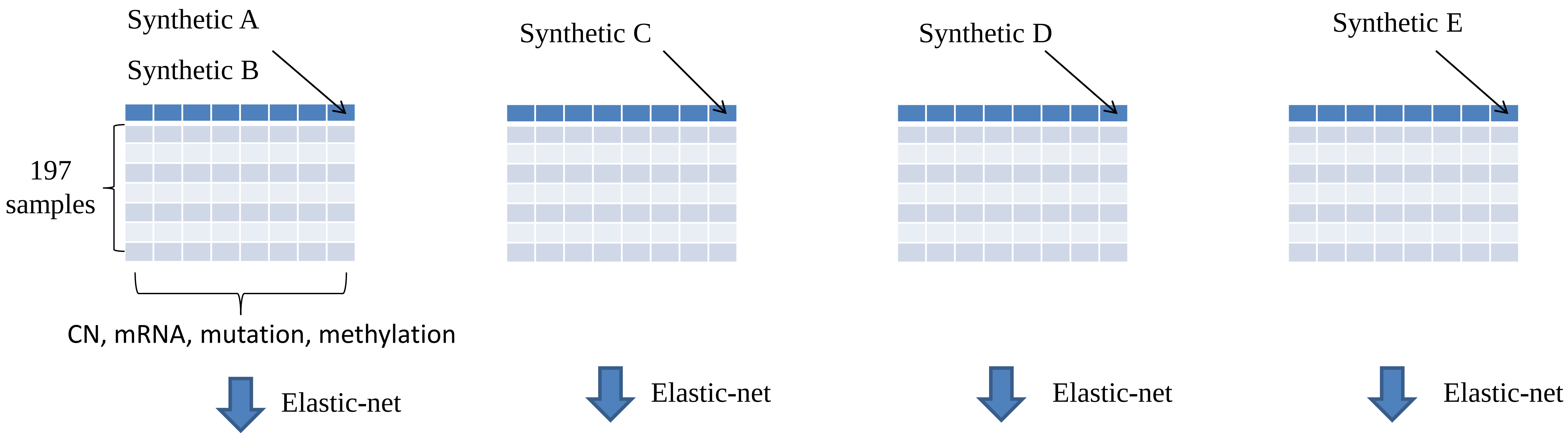

Rank	Genetic feature
1	*Synthetic A*
2	*Synthetic B*
3	*Gene 1*

Rank	Genetic feature
1	*Synthetic C*
2	*Gene 1*
3	*Gene 2*

Rank	Genetic feature
1	*Synthetic D*
2	*Gene 1*
3	*Gene 2*

Rank	Genetic feature
1	*Synthetic E*
2	*Gene 1*
3	*Gene 2*
